# External Quality Assessment (EQA) for Molecular Diagnostics of Zika Virus: Experiences from an International EQA Programme, 2016–2018

**DOI:** 10.3390/v10090491

**Published:** 2018-09-13

**Authors:** Oliver Donoso Mantke, Elaine McCulloch, Paul S. Wallace, Constanze Yue, Sally A. Baylis, Matthias Niedrig

**Affiliations:** 1 Quality Control for Molecular Diagnostics (QCMD), Unit 5, Technology Terrace, Todd Campus, West of Scotland Science Park, Glasgow G20 0XA, UK; ElaineMcCulloch@qcmd.org (E.M.); PaulWallace@qcmd.org (P.S.W.); 2 Division of Virology, Paul-Ehrlich-Institut (PEI), Federal Institute for Vaccines and Biomedicines, 63225 Langen, Germany; Constanze.Yue@pei.de (C.Y.); Sally.Baylis@pei.de (S.A.B.); 3 Robert Koch-Institut (RKI), 13353 Berlin, Germany; niedrigm@rki.de

**Keywords:** Zika virus, mosquito-borne flavivirus, emerging arbovirus, outbreak control, molecular diagnostics, laboratory preparedness, assay standardization, external quality assessment, EQA, QCMD

## Abstract

Quality Control for Molecular Diagnostics (QCMD), an international provider for External Quality Assessment (EQA) programmes, has introduced a programme for molecular diagnostics of Zika virus (ZIKV) in 2016, which has been continuously offered to interested laboratories since that time. The EQA schemes provided from 2016 to 2018 revealed that 86.7% (92/106), 82.4% (89/108), and 88.2% (90/102) of the participating laboratories reported correct results for all samples, respectively in 2016, 2017, and 2018. The review of results indicated a need for improvement concerning analytical sensitivity and specificity of the test methods. Comparison with the outcomes of other EQA initiatives briefly summarized here show that continuous quality assurance is important to improve laboratory performance and to increase preparedness with reliable diagnostic assays for effective patient management, infection and outbreak control.

## 1. Introduction

Zika virus (ZIKV) is a mosquito-borne flavivirus which had been considered to have low pathogenicity, with only very sporadic human cases and outbreaks reported in the past [1]. In 2015, ZIKV was first detected in patients in Brazil, which was a viral strain of the Asian lineage [2,3]. After introduction into South America, the virus has spread rapidly in over 48 countries and territories in the Americas, with over 220,000 confirmed autochthonous cases and over 3500 confirmed congenital cases associated with ZIKV infection [4]. During this rapid emergence, the World Health Organization (WHO) declared a Public Health Emergency of International Concern (PHEIC) in response to a cluster of microcephaly cases among newborns and other neurological disorders, like Guillain-Barré syndrome, in adults [5]. In accordance with the situation, the WHO assigned the development, assessment and validation of ZIKV diagnostic assays as a priority [6]. The PHEIC declaration ended in November 2016 with a longer-term programmatic approach for control and prevention [7].

Laboratory preparedness with access to reliable diagnostic assays, when facing an outbreak of arboviral infections and increase of international travel, is a key issue for patient management, infection and outbreak control worldwide [8,9,10,11,12,13]. Since the beginning of the ZIKV outbreak many diagnostic tests, mostly molecular in-house assays, have been developed [6,14,15,16]. Molecular methods are preferred for laboratory diagnosis of acute cases, especially in the early stage of arboviral infection [17]. Specific assays have been published for Asian and African ZIKV strains targeting several genomic regions including the envelope and NS5 [14,15,17]. Several months after the initial outbreak, there are also some commercial molecular assays available for ZIKV genome detection which have received regulatory approval [6,18,19].

Quality Control for Molecular Diagnostics (QCMD) is an international provider for External Quality Assessment (EQA) programmes covering a comprehensive range of infectious diseases [20]. The aims of QCMD’s EQA programmes are to help monitor and improve laboratory quality by assessing a laboratory’s use of molecular diagnostic technologies within the routine clinical setting. Participation in EQA programmes is a requirement for achieving accreditation/certification according to the International Organization for Standardization (ISO) 15189 (‘Medical laboratories–requirements for quality and competence’ [21]) or equivalent regulatory requirements. After the declaration of the PHEIC related to ZIKV in February 2016, QCMD rapidly introduced an EQA programme for molecular diagnostics of ZIKV in August 2016 to support the laboratory’s requirements and provide appropriate proficiency testing options from then on. Here we review the results of the EQA schemes performed from 2016 to 2018 showing need for improvement concerning analytical sensitivity and specificity of the test methods.

## 2. The QCMD Zika Virus EQA Programme, 2016–2018

QCMD distributes the EQA programme for molecular diagnostics of ZIKV to registered laboratories annually. The annual EQA schemes have different panel compositions comprising eight to 12 lyophilized samples with samples containing cell culture-derived, inactivated African- and Asian-lineage ZIKV at different concentrations, specificity controls covering a range of related flaviviruses and other arboviruses, and negative samples with transport medium only. Furthermore, each QCMD panel includes the first WHO international standard (IS) for ZIKV for molecular assays, developed by the Paul-Ehrlich-Institut (PEI code number 11468/16 [22]) which allows traceability of EQA materials and comparability of laboratory performance in order to resolve the lack of assay standardization [23]. This material was officially established as WHO IS by the WHO Expert Committee on Biological Standardization in October 2016, however the candidate material had been made available prior to its establishment as the WHO IS for proficiency testing, assay development and clinical use because of the urgent need for quality-assured diagnostics [6].

All EQA programme and panel design specifications are defined by QCMD in close collaboration with Scientific Experts in the field, based on epidemiological, clinical and/or technical aspects. Participating laboratories are expected to test each panel using their routine molecular assays and workflows, and to report their results together with information of the applied extraction and amplification methods through the online reporting system to QCMD [20]. 

The panel compositions and performances for 2016–2018 are shown in Table 1. Participation has been relatively high since the introduction of the EQA programme, with 106 (116 datasets), 108 (114 datasets), and 102 (112 datasets) participating laboratories from around 37 countries worldwide, including those in affected geographical regions (with up to 17% of participating laboratories from affected countries like Brazil, Mexico, Panama), respectively, in 2016, 2017 and 2018. The results were predominantly generated by commercial assays with 54.3% (2016), 57.0% (2017), and 52.7% (2018) of the submitted datasets. No remarkable difference in performance between in-house and commercial assays were observed, however for some specific commercial assays analytical challenges could be identified concerning sensitivity with lower sample concentrations as well as specificity issues producing false positive results (data not specified). Please note that these observations do not allow a solid and statistically assured statement as EQA studies per se are not appropriate for assay validation.

In the EQA schemes of 2016–2018, all samples were designated by the QCMD Scientific Experts as Core proficiency samples, which are expected to be correctly reported by the participating laboratories within the EQA challenge in order to show an acceptable level of proficiency/successful participation. The percentage of datasets with All Core samples correctly reported were 86.2% (2016), 82.5% (2017), and 85.7% (2018) (see Table 1). At the laboratory level, these were 86.7% (92/106), 82.4% (89/108), and 88.2% (90/102), respectively. Incorrect results (including false positive or not determined) were reported for the true negative sample containing transport medium only (2.6% in 2016; 3.5% in 2017; 0.0% in 2018), for a specificity negative sample containing a mixture of flaviviruses other than ZIKV (5.2% in 2016; 1.8% in 2017; 1.8% in 2018), and for a specificity negative sample containing chikungunya virus (3.4%, only included in 2016).

Review of the three QCMD EQA schemes performed between 2016 and 2018 demonstrate that the overall qualitative performance of participating laboratories for molecular diagnostics of ZIKV was at an acceptable level. However, analytical sensitivity and specificity remain a challenge. False positive results may lead to misdiagnosis and potentially affect clinical decisions. Processes should be reviewed by laboratories reporting false positive results. The risk of a false negative result was a concern for a small number of laboratories and may result in failure to detect a ZIKV in low concentration in infected patients during the acute phase of the disease. Laboratories who were unable to detect the lower concentrated samples should strive to improve the sensitivity of the ZIKV molecular assay used.

## 3. Review of Results from Other EQA Initiatives

To date, a further three EQA activities for ZIKV molecular diagnostics performed by different groups have been identified showing similar observations as mentioned above [26,27,28].

Fischer et al. [26] conducted an EQA study for Brazilian laboratories in 2017. Fifteen laboratories participated in this study applying their routine molecular diagnostics for ZIKV on a panel comprising 12 lyophilized samples with inactivated full virus spiked into arbovirus-negative human plasma. The panel included four ZIKV-positive samples with 10^3^–10^6^ RNA copies/mL (representing the African and Asian lineage, including the outbreak strain in the Americas) to assess sensitivity, seven different arbovirus-positive samples (chikungunya virus, CHIKV; dengue viruses, DENV-2 & DENV-4; Japanese encephalitis virus, JEV; St. Louis encephalitis virus, SLEV; West Nile virus, WNV; yellow fever virus, YFV) for specificity control, and a negative plasma sample. In addition, the WHO IS [24] as well as a ZIKV armored RNA standard available at the EVAg portal (European Virus Archive goes Global) were provided as references for quantification of the ZIKV-positive samples. Only 27.0% (four of 15 laboratories) reported correct results for all samples, while 73.0% (11/15) had limited sensitivity (correctly testing only the two samples with the highest ZIKV concentration) and/or specificity (including false positive results from ZIKV-negative samples). 

Abdad et al. [27] pointed out how important it is to continually offer and expand an EQA initiative for emerging arboviruses which helps to assess the laboratory performance and to increase the preparedness in a certain region. The WHO Regional Office for the Western Pacific Region (WPR) started with an EQA pilot for two participating laboratories in 2011, including only dengue virus, and developed it to a global EQA programme for arbovirus diagnostics (including CHIKV, DENV, YFV, and ZIKV) with participation of 96 laboratories worldwide (of which 25 were coming from 19 countries in the WPR) in 2016. In 2016, the panels contained blinded samples with different concentrations for the four targeted arboviruses and were shipped to the participants between November and December. For ZIKV, 72.0% (18 of 25 laboratories of the WPR) reported correct results in all samples, while 28.0% (seven) had at least one false result which was not further specified.

Charrel et al. [28] published an EQA study that was performed by the Emerging Viral Diseases Expert Laboratory Network (EVD-LabNet, Rotterdam, The Netherlands) in October/November 2016 to assess and to improve the capability of European reference and expert laboratories for ZIKV molecular detection. Fifty laboratories (reporting 85 datasets) from 31 countries took part in this study, using mostly in-house assays (72.0% of the submitted datasets). The panel consisted of 12 lyophilized samples with inactivated virus spiked into plasma or urine, including six ZIKV-positive samples with 10^3^–10^9^ copies/mL (representing the African and Asian lineage, including the outbreak strain in the Americas; calibrated using in vitro-transcribed ZIKV RNA), three different arbovirus-positive samples (CHIKV, YFV, DENV-1), and three negative plasma or urine samples. Based on the submitted results, all samples were assigned as core samples, having a ZIKV status scored correctly by >50% of the participating laboratories. The study revealed that only 40.0% of the laboratories (20/50), representing 45.0% of the countries, achieved a sufficient analytical performance. 60.0% of the laboratories (30/50) had a need to improve their molecular ZIKV detection in relation with specificity, but more obviously with sensitivity as only half of the laboratories correctly scored the sample with the lowest concentration.

## 4. Conclusions

Although the comparison of the recent results from the QCMD Zika Virus EQA Programme done for 2016–2018 with the briefly summarized outcomes of the other EQA initiatives for ZIKV molecular diagnostics shows great variations in laboratory performance with 27.0–88.2% of participating laboratories reporting correct results for all samples, all EQAs indicate that there is a clear need to improve sensitivity and specificity of ZIKV molecular assays.

The ZIKV molecular diagnostics would benefit greatly from the use of standards and controls in order to ensure that laboratories would also detect lower viral loads as known from the literature [17,29,30] to avoid false negative results during acute infection. The continued use of the WHO IS as included in the QCMD EQA programme for molecular diagnostics of ZIKV will help to support assay standardization and to improve quality performance of molecular ZIKV diagnostics.

As ZIKV and other arboviruses (e.g., DENV, CHIKV) co-circulate affecting many endemic countries and at-risk countries globally, being a risk for local populations and international travelers, it is important that laboratories can accurately detect and differentiate to avoid false positive results, potentially having serious consequences for patients as exemplified by a dramatic increase in abortion requests in South America during the 2016 ZIKV outbreak [31]. Regular participation in EQA schemes by laboratories is important to monitor the improvement in laboratory performance; also, for laboratories outside endemic regions a regular EQA participation would provide confidence in tests when positive samples are not routinely seen.

Laboratories should be aware of the limitation of their assays and perform their own validation and verification in line with ISO 15189 and other requirements. Continually offered EQA programmes for arbovirus diagnostics are an important tool to improve laboratory performance and to increase preparedness having quality-assured assays disponible for effective patient management, infection and outbreak control.

## Figures and Tables

**Table 1:** Quality Control for Molecular Diagnostics (QCMD) Zika Virus External Quality Assessment (EQA) Programme. Panel compositions and performances, 2016–2018.

**(A) Data and Results on the Individual EQA Sample Level**										
		**EQA Scheme 2016**			**EQA Scheme 2017**			**EQA Scheme 2018**		
**Sample Content**	**Target Concentration [log_10_ IU/mL]**	**Assigned as Sample**	**Percentage Correct [%]**	**Datasets ^#^ (*n*)**	**Assigned as Sample**	**Percentage Correct [%]**	**Datasets ^#^ (*n*)**	**Assigned as Sample**	**Percentage Correct [%]**	**Datasets ^#^ (*n*)**
ZIKV MR766	4.7	ZIKA16-08	97.4	116	ZIKA17S-07	97.4	114	ZIKA18S-05	97.3	112
ZIKV MR766	4.7	ZIKA16-09	99.1	116	-	-	-	-	-	-
ZIKV MR766	3.7	ZIKA16-10	95.7	116	ZIKA17S-06	95.6	114	ZIKA18S-04	95.5	112
ZIKV MR766	2.7	ZIKA16-02	93.1	116	ZIKA17S-03	89.5	114	ZIKA18S-06	89.3	112
ZIKV 11474/16	5.7	ZIKA16-04	100	116	-	-	-	-	-	-
ZIKV 11474/16	3.7	-	-	-	ZIKA17S-01	94.7	114	ZIKA18S-01	93.8	112
ZIKV 11468/16	5.7	ZIKA16-06	100	116	-	-	-	-	-	-
ZIKV 11468/16	4.7	ZIKA16-03	100	116	ZIKA17S-05	99.1	114	ZIKA18S-03	97.3	112
ZIKV 11468/16	3.7	-	-	-	ZIKA17S-04	96.5	114	ZIKA18S-02	94.6	112
Non-Zika flaviviruses	-	ZIKA16-01	94.8	116	ZIKA17S-02	98.2	114	ZIKA18S-07	98.2	112
Chikungunya virus	-	ZIKA16-07	96.6	116	-	-	-	-	-	-
Transport medium	-	ZIKA16-05	97.4	116	ZIKA17S-08	96.5	114	ZIKA18S-08	100	112
**(B) Overall Qualitative Performance for the Core Sample Panel**										
			**EQA Scheme 2016**			**EQA Scheme 2017**			**EQA Scheme 2018**	
Percentage [%] of datasets with All Core samples correct			86.2 (10/10 core samples)	116 datasets ^#^		82.5 (8/8 core samples)	114 datasets ^#^		85.7 (8/8 core samples)	112 datasets ^#^

Positive samples contained different concentrations of Zika virus strain Uganda MR766 provided by the Robert Koch-Institut (RKI) (representing the African lineage), ZIKV reference material 11474/16 prepared by the Paul-Ehrlich-Institut (PEI) (from French Polynesian ZIKV strain PF13/251013-18, representing the Asian lineage), or WHO IS preparation 11468/16 developed by PEI (from French Polynesian ZIKV strain PF13/251013-18, representing the Asian lineage) [24,25]. Negative samples contained a mixed sample of flaviviruses other than ZIKV (dengue virus serotype 2, DENV-2; West Nile virus strain NY99, WNV-NY99 and yellow fever virus 17D, YFV-17D) and/or a sample with chikungunya virus (CHIKV) for specificity control, and a sample with transport medium only (which was used as sample matrix for the EQA samples). All cell culture-derived arboviral samples were inactivated and lyophilized. ^#^ A dataset is defined as one qualitative laboratory result per sample and applied workflow.
